# Synthesis and crystal structure of a new hybrid organic–inorganic material containing neutral mol­ecules, cations and hepta­molybdate anions

**DOI:** 10.1107/S2056989019008454

**Published:** 2019-06-21

**Authors:** Bougar Sarr, Abdou Mbaye, Wally Diallo, Cheikh Abdoul Khadir Diop, Mamadou Sidibe, Francois Michaud

**Affiliations:** a Laboratoire de Chimie Minérale et Analytique, Département de Chimie, Faculté des Sciences et Téchniques, Université Cheikh Anta Diop, Dakar, Senegal; b Laboratoire de Chimie et de Physique des Matériaux (LCPM) de l’Université Assane Seck de Ziguinchor (UASZ), BP 523 Ziguinchor, Senegal; c Service Commun d’Analyse par Diffraction des Rayons X, Universite de Bretagne Occidentale, 6, avenue Victor Le Gorgeu, CS 93837, F-29238 Brest cedex 3, France

**Keywords:** crystal structure, polyoxomolybdates, IR spectroscopic study, hydrogen bonding

## Abstract

The title compound, hexa­kis­(2-methyl-1*H*-imidazol-3-ium) hepta­molybdate 2-methyl-1*H*-imidazole disolvate dihydrate, (C_4_H_7_N_2_)_6_[Mo_7_O_24_]·2C_4_H_6_N_2_·2H_2_O, was prepared from 2-methyl­imidazole and ammonium hepta­molybdate tetra­hydrate in acid solution. The [Mo_7_O_24_]^6−^ hepta­molybdate cluster anion is accompanied by six protonated (C_4_H_7_N_2_)^+^ 2-methyl­imidazolium cations, two neutral C_4_H_6_N_2_ 2-methyl­imidazole mol­ecules and two water mol­ecules of crystallization. The cluster consists of seven distorted MoO_6_ octa­hedra sharing edges or vertices. In the crystal, the components are linked by N—H⋯N, N—H⋯O, O—H⋯O, N—H⋯(O,O) and O—H⋯(O,O) hydrogen bonds, generating a three-dimensional network. Weak C—H⋯O inter­actions consolidate the packing.

## Abstract 2

In the crystal structure of hexa­kis­(2-methyl-1*H*-imidazol-3-ium) hepta­molybdate 2-methyl-1*H*-imidazole disolvate dihydrate,(C_4_H_7_N_2_)_6_[Mo_7_O_24_]·2C_4_H_6_N_2_·2H_2_O, [Mo_7_O_24_]^6−^ hepta­molybdate anions, 2-methyl­imidazolium cations, neutral 2-methyl­imidazole mol­ecules and water mol­ecules are linked through hydrogen bonds into a three-dimensional network.

## Chemical context   

Polyoxometalates (POMs) are clusters of transition metals (*M* = V, Nb, Ta, Mo, W, ⋯) and oxygen atoms with a structural and compositional diversity that lead to numerous applications because of their electrochemical, optical, catalytic and photochromic properties as well as their anti­viral and anti­tumor activities (Katsoulis, 1998 ▸; Hasenknopf, 2005 ▸; Gerth *et al.*, 2005 ▸; Coué *et al.*, 2007 ▸). In this context, the [Mo_7_O_24_]^6−^ hepta­molybdate anion has been isolated with numerous different counter-cations such as 4-amino­pyridinium, *N*-pentyl­ammonium, di­ethyl­enetri­ammonium, *N*,*N*,*N*′,*N*′-tetra­methyl­ethylenedi­ammonium, tetra­methyl­ammonium, guanidinium, hexa­nedi­ammonium, butan-1-aminium, ammonium, potassium and sodium (Román *et al.*, 1985 ▸, 1988 ▸, 1990 ▸, 1992 ▸; Don & Weakly, 1981 ▸; Gatehouse & Leverett, 1968 ▸; Sjöbom & Hedman, 1973 ▸; Niu *et al.*, 1996 ▸; Himeno *et al.*, 1997 ▸; Reinoso *et al.*, 2008 ▸; Ftini, 2015 ▸; Khandolkar *et al.*, 2016 ▸). As a contin­uation of our work in this area (Sarr *et al.*, 2018 ▸), we now describe the synthesis and structure of the title compound (I), which is notable for the incorporation of both protonated [C_4_H_7_N_2_]^+^ 2-methyl­imidazolium cations and neutral C_4_H_6_N_2_ 2-methyl­imidazole mol­ecules in the crystal.

## Structural commentary   

**Figure 1:**
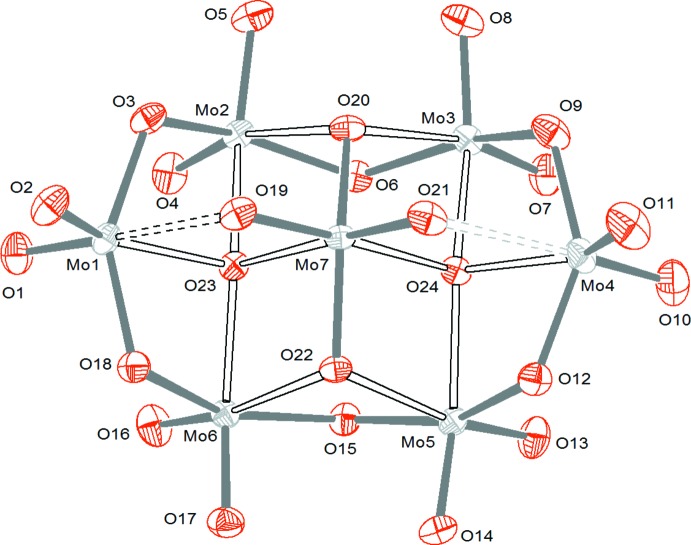
Mol­ecular structure of the [Mo_7_O_24_]^6−^ hepta­molybdate cluster anion in (I).

The title compound is characterized by the presence of the familiar [Mo_7_O_24_]^6−^ hepta­molybdate cluster anion (Fig. 1 ▸).
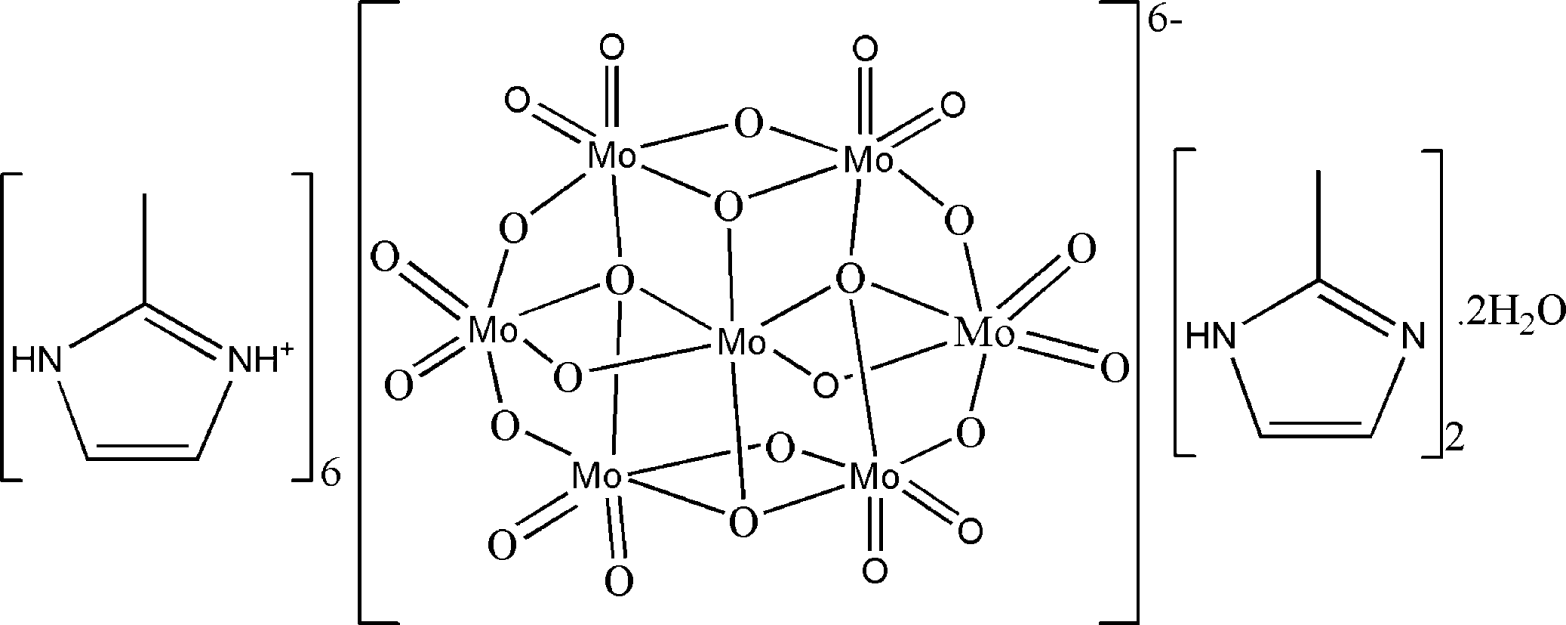


**Figure 2:**
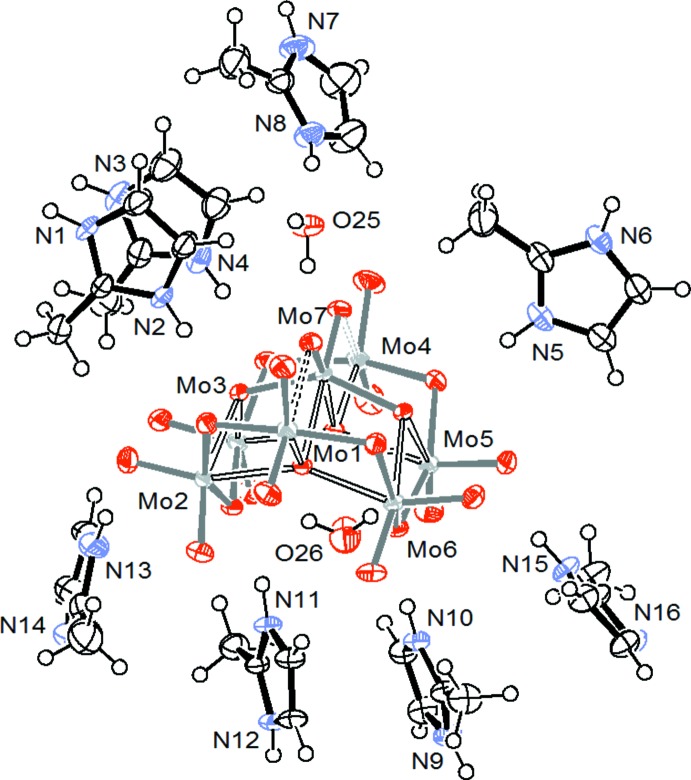
The asymmetric unit of (I) with displacement ellipsoids drawn at the 50% probability level.

There are four categories of oxygen atoms within the polyanion: O_t_ (terminal oxygen atoms), μ^2^-O (oxygen atoms bridging two molybdenum atoms), μ^3^-O (oxygen atoms bridging three molybdenum atoms) and μ^4^-O (oxygen atoms bridging four molybdenum atoms). All of the Mo atoms are bound to two terminal oxygen atoms except for Mo7, which is located in the ‘core’ of the cluster. The geometrical data for the cluster in (I) are consistent with those found in previous studies (Román *et al.*, 1992 ▸; Reinoso *et al.*, 2008 ▸): the Mo—O bond lengths vary between 1.707 (2) and 1.726 (2) Å for O_t_, 1.754 (2)—2.453 (2) Å for μ^2^-O, 1.8945 (19)–2.3057 (19) Å for μ^3^-O and 2.1329 (19)–2.3011 (18) Å for μ^4^-O. The variations of Mo—O bond lengths and O—Mo—O angles indicate that all seven octa­hedra (MoO_6_) within the cluster are highly distorted. As in the compound (H_3_dien)_2_[Mo_7_O_24_]·4H_2_O (Román *et al.*, 1988 ▸), we note that the longest Mo—O bond length derives from an oxygen atom bridging two molybdenum atoms (μ^2^-O). As well as the [Mo_7_O_24_]^6−^ anion, six (C_4_H_7_N_2_)^+^ cations, two neutral C_4_H_6_N_2_ mol­ecules and two water mol­ecules of crystallization are present in the asymmetric unit (Fig. 2 ▸).

## Supra­molecular features   

**Figure 3:**
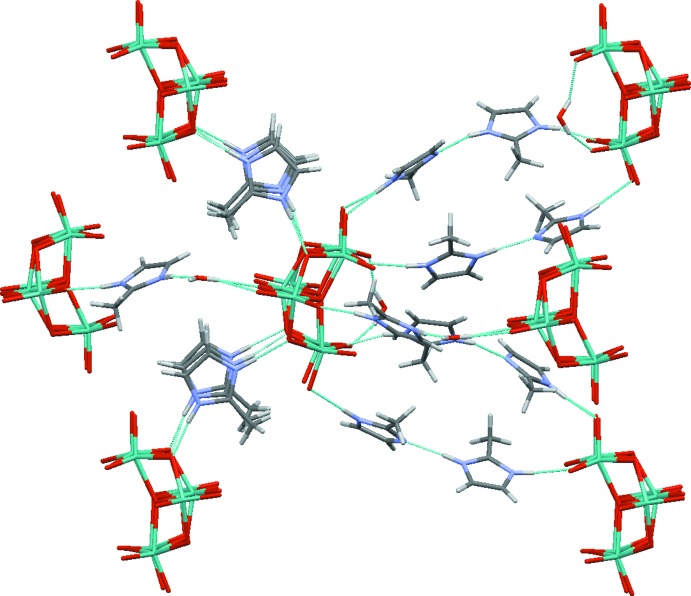
Detail of the structure of (I) showing the inter­connections of the [Mo_7_O_24_]^6−^ anion with its neighbours.

In the crystal, each hepta­molybdate anion inter­acts with six neighbours *via* the water mol­ecules, (C_4_H_7_N_2_)^+^ cations and/or neutral 2-methyl­imidazole mol­ecules (Fig. 3 ▸). These inter­actions occur through simple O—H⋯O, N—H⋯O and N—H⋯N and bifurcated N—H⋯(O,O) and O—H⋯(O,O) hydrogen bonds (Table 1 ▸) involving three categories of oxygen atoms of the polyanion: O_t_, μ^2^-O and μ^3^-O. The N—H⋯N hydrogen bonds from N9 and N12 link (C_4_H_7_N_2_)^+^ cations to neutral mol­ecules. The packing is consolidated by weak C—H⋯O links (Table 1 ▸). The overall hydrogen-bonding topology is an infinite three-dimensional network.

## Database survey   

A search of the Cambridge Structural Database (CSD, version 5.40, update November 2018; Groom *et al.*, 2016 ▸) resulted in 35 hits for the hepta­molybdate anion and 90 hits for the 2-methyl­imidazolium cation.

## Synthesis and crystallization   

Sulfuric acid (2.1 g, 21.7 mmol), 2-methyl­imidazole (3.5 g, 43.4 mmol) and ammonium hepta­molybdate tetra­hydrate (2.2 g, 1.8 mmol) in a ratio of 1:2:1/12 were dissolved in water (60 ml). The solution was stirred for one h and evaporated in the oven at 333 K to yield a whitish precipitate. The precipitate was recrystallized from methanol solution: after two weeks at room temperature, colourless prisms of (I) were recovered.

The IR spectrum of (I) is included in the supporting information. The absorption bands at 3400 and 3395 cm^−1^ corres­pond to ν(O—H) stretches and indicate the presence of water mol­ecules and those at 1621 and 1564 cm^−1^ to the deformation vibrations δ(O—H). The bands centered at 3132 and 1431 cm^−1^ with shoulders are respectively attributed to the stretching and deformation vibrations of the N—H bonds of the protonated and/or non-protonated entities of 2-methyl­imidazole (Jinnah *et al.*, 2004 ▸). The bands between 2904–2686 cm^−1^ are attributed to the stretching vibrations of the C—H bonds, while that at 1291 cm^−1^ is a δ(C—H) deformation vibration (Jinnah *et al.*, 2004 ▸). The two bands at 929 and 900 cm^−1^ correspond to ν(Mo—Ot) stretching vibrations while the bands between 838 and 650 cm^−1^ are typical for the vibrations of ν(Mo—O—Mo) and ν[Mo—(μ-O)] groupings (Dey *et al.*, 2011 ▸).

## Refinement details   

Crystal data, data collection and structure refinement details are summarized in Table 2 ▸. All H atoms treated by a mixture of independent and constrained refinement were placed in geometrically idealized positions and constrained to ride on their parent atoms, with N—H distances of 0.87 (2), 0.88 (2) and 0.89 (2) Å, Cmeth­yl—H = 0.97/0.98 Å and Cmethine—H = 0.94/0.95 Å, and with *U*_iso_ (H) = 1.2*U*_eq_(C,N) or 1.5*U*_eq_(C-meth­yl).

## Supplementary Material

Crystal structure: contains datablock(s) global, I. DOI: 10.1107/S2056989019008454/hb7831sup1.cif

Structure factors: contains datablock(s) I. DOI: 10.1107/S2056989019008454/hb7831Isup2.hkl

The IR spectrum of compound (I). DOI: 10.1107/S2056989019008454/hb7831sup3.tif

CCDC reference: 1922927

Additional supporting information:  crystallographic information; 3D view; checkCIF report

## Figures and Tables

**Table 1:** Hydrogen-bond geometry (Å, °)

*D*—H⋯*A*	*D*—H	H⋯*A*	*D*⋯*A*	*D*—H⋯*A*
N1—H1*N*⋯O22^i^	0.88 (2)	1.85 (2)	2.710 (3)	167 (2)
N2—H2*N*⋯O20	0.87 (2)	1.79 (2)	2.655 (3)	172 (2)
N3—H3*N*⋯O18^i^	0.88 (2)	1.87 (2)	2.743 (4)	171 (5)
N4—H4*N*⋯O9	0.88 (2)	1.79 (2)	2.662 (4)	171 (5)
N5—H5*N*⋯O12	0.89 (2)	1.77 (3)	2.644 (4)	168 (4)
N6—H6*N*⋯O3^ii^	0.90 (2)	1.80 (3)	2.684 (4)	168 (5)
N7—H7*N*⋯O1^iii^	0.89 (3)	1.83 (3)	2.697 (4)	167 (3)
N8—H8*N*⋯O25	0.89 (3)	1.78 (3)	2.659 (4)	172 (5)
N9—H9*N*⋯N14^iv^	0.89 (2)	1.81 (2)	2.698 (4)	177 (3)
N10—H10*N*⋯O15	0.88 (2)	1.98 (2)	2.852 (3)	174 (3)
N11—H11*N*⋯O6	0.88 (2)	1.78 (2)	2.636 (3)	166 (3)
N12—H12*N*⋯N16^v^	0.88 (2)	1.87 (2)	2.725 (4)	165 (3)
N13—H13*N*⋯O5	0.88 (4)	2.32 (4)	3.186 (5)	167 (3)
N13—H13*N*⋯O8	0.88 (4)	2.55 (4)	3.043 (5)	116 (2)
N15—H15*N*⋯O14	0.87 (3)	2.22 (3)	2.953 (4)	142 (3)
N15—H15*N*⋯O17	0.87 (3)	2.27 (3)	2.917 (4)	132 (3)
O25—H25*V*⋯O19	0.86 (2)	2.00 (2)	2.788 (3)	154 (5)
O25—H25*W*⋯O26^vi^	0.86 (3)	1.85 (3)	2.702 (4)	176 (5)
O26—H26*V*⋯O10	0.83 (4)	2.50 (6)	2.999 (4)	119 (5)
O26—H26*V*⋯O13	0.83 (4)	2.25 (5)	3.009 (4)	151 (6)
O26—H26*W*⋯O7	0.83 (4)	1.99 (6)	2.737 (4)	149 (6)
C4—H4*A*⋯O8	0.98	2.35	3.228 (5)	149
C8—H8*B*⋯O2^i^	0.98	2.54	3.517 (6)	176
C8—H8*C*⋯O8	0.98	2.49	3.386 (5)	152
C10—H10⋯O26^vii^	0.95	2.45	3.318 (5)	151
C12—H12*A*⋯O5^ii^	0.98	2.44	3.407 (5)	167
C13—H13⋯O4^iii^	0.95	2.50	3.297 (6)	141
C17—H17⋯O2^viii^	0.95	2.46	3.154 (4)	130
C18—H18⋯O26	0.95	2.58	3.522 (5)	172
C21—H21⋯O16	0.95	2.46	3.302 (4)	147
C22—H22⋯O11^ix^	0.95	2.22	3.113 (4)	156
C24—H24*B*⋯O7	0.98	2.50	3.346 (5)	145
C25—H25⋯O8	0.95	2.59	3.055 (5)	110
C32—H32*B*⋯O14	0.98	2.55	3.234 (5)	126

Symmetry codes: (i) 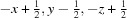; (ii) 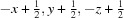; (iii) 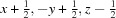; (iv) 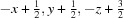; (v) 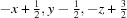; (vi) 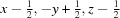; (vii) 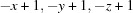; (viii) 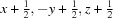; (ix) 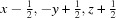.

**Table 2:** Experimental details

Crystal data	
Chemical formula	(C_4_H_7_N_2_)_6_[Mo_7_O_24_]·2C_4_H_6_N_2_·2H_2_O
*M*_r_	1754.52
Crystal system, space group	Monoclinic, *P*2_1_/*n*
Temperature (K)	170
*a*, *b*, *c* (Å)	16.5325 (2), 17.5842 (2), 19.8873 (2)
β (°)	90.653 (1)
*V* (Å^3^)	5781.08 (11)
*Z*	4
Radiation type	Mo *K*α
μ (mm^−1^)	1.56
Crystal size (mm)	0.38 × 0.28 × 0.19

Data collection	
Diffractometer	Agilent Xcalibur, Sapphire2
Absorption correction	Multi-scan (*CrysAlis PRO*; Agilent, 2014 ▸)
*T*_min_, *T*_max_	0.476, 0.756
No. of measured, independent and observed [*I* > 2σ(*I*)] reflections	104921, 27993, 21593
*R*_int_	0.053
(sin θ/λ)_max_ (Å^−1^)	0.833

Refinement	
*R*[*F* ^2^ > 2σ(*F* ^2^)], *wR*(*F* ^2^), *S*	0.046, 0.120, 1.15
No. of reflections	27993
No. of parameters	792
No. of restraints	58
H-atom treatment	H atoms treated by a mixture of independent and constrained refinement
Δρ_max_, Δρ_min_ (e Å^−3^)	1.83, −2.87

Computer programs: *CrysAlis PRO* (Agilent, 2014 ▸), *SIR92* (Altomare *et al.*, 1992 ▸), *SHELXL97* (Sheldrick, 2008 ▸), *ORTEP-3 for Windows* and *WinGX* (Farrugia, 2012 ▸), *Mercury* (Macrae *et al.*, 2008 ▸) and *PLATON* (Spek, 2009 ▸).

## supplementary crystallographic information

### Crystal data

**Table:**

(C_4_H_7_N_2_)_6_[Mo_7_O_24_]·2C_4_H_6_N_2_·2H_2_O	*F*(000) = 3456
*M**_r_* = 1754.52	*D*_x_ = 2.016 Mg m^−^^3^
Monoclinic, *P*2_1_/*n*	Mo *K*α radiation, λ = 0.71073 Å
Hall symbol: -P 2yn	Cell parameters from 38522 reflections
*a* = 16.5325 (2) Å	θ = 3.5–37.4°
*b* = 17.5842 (2) Å	µ = 1.56 mm^−^^1^
*c* = 19.8873 (2) Å	*T* = 170 K
β = 90.653 (1)°	Fragment of prism, colourless
*V* = 5781.08 (11) Å^3^	0.38 × 0.28 × 0.19 mm
*Z* = 4	

### Data collection

**Table:**

Agilent Xcalibur, Sapphire2, large Be window diffractometer	27993 independent reflections
Radiation source: Enhance (Mo) X-ray Source	21593 reflections with *I* > 2σ(*I*)
Graphite monochromator	*R*_int_ = 0.053
Detector resolution: 8.3622 pixels mm^-1^	θ_max_ = 36.3°, θ_min_ = 3.3°
ω scans	*h* = −24→27
Absorption correction: multi-scan (CrysAlis PRO; Agilent, 2014)	*k* = −29→29
*T*_min_ = 0.476, *T*_max_ = 0.756	*l* = −33→33
104921 measured reflections	

### Refinement

**Table:**

Refinement on *F*^2^	Primary atom site location: structure-invariant direct methods
Least-squares matrix: full	Secondary atom site location: difference Fourier map
*R*[*F*^2^ > 2σ(*F*^2^)] = 0.046	Hydrogen site location: inferred from neighbouring sites
*wR*(*F*^2^) = 0.120	H atoms treated by a mixture of independent and constrained refinement
*S* = 1.15	*w* = 1/[σ^2^(*F*_o_^2^) + (0.0397*P*)^2^ + 11.2859*P*] where *P* = (*F*_o_^2^ + 2*F*_c_^2^)/3
27993 reflections	(Δ/σ)_max_ = 0.011
792 parameters	Δρ_max_ = 1.83 e Å^−^^3^
58 restraints	Δρ_min_ = −2.87 e Å^−^^3^

### Special details

**Table:**

Geometry. All s.u.'s (except the s.u. in the dihedral angle between two l.s. planes) are estimated using the full covariance matrix. The cell s.u.'s are taken into account individually in the estimation of s.u.'s in distances, angles and torsion angles; correlations between s.u.'s in cell parameters are only used when they are defined by crystal symmetry. An approximate (isotropic) treatment of cell s.u.'s is used for estimating s.u.'s involving l.s. planes.
Refinement. Refinement of F^2^ against ALL reflections. The weighted R-factor wR and goodness of fit S are based on F^2^, conventional R-factors R are based on F, with F set to zero for negative F^2^. The threshold expression of F^2^ > 2σ(F^2^) is used only for calculating R-factors(gt) etc. and is not relevant to the choice of reflections for refinement. R-factors based on F^2^ are statistically about twice as large as those based on F, and R- factors based on ALL data will be even larger.

### Fractional atomic coordinates and isotropic or equivalent isotropic displacement parameters (Å^2^)

**Table:**

	*x*	*y*	*z*	*U*_iso_*/*U*_eq_	
O1	−0.04305 (13)	0.24793 (14)	0.39565 (12)	0.0280 (4)	
O2	0.00551 (14)	0.27946 (14)	0.26843 (11)	0.0284 (5)	
O3	0.07596 (13)	0.15830 (12)	0.34241 (10)	0.0230 (4)	
O4	0.07336 (14)	0.12579 (14)	0.48407 (11)	0.0269 (4)	
O5	0.15457 (15)	0.03530 (13)	0.39571 (12)	0.0295 (5)	
O6	0.23807 (12)	0.13563 (11)	0.47918 (9)	0.0185 (3)	
O7	0.40034 (14)	0.12283 (14)	0.50582 (12)	0.0284 (5)	
O8	0.33831 (15)	0.04054 (13)	0.40157 (12)	0.0280 (4)	
O9	0.42573 (14)	0.16391 (13)	0.36926 (12)	0.0268 (4)	
O10	0.52563 (15)	0.25984 (16)	0.44633 (15)	0.0364 (6)	
O11	0.50709 (16)	0.28585 (17)	0.31122 (14)	0.0393 (6)	
O12	0.41549 (13)	0.37036 (12)	0.40412 (11)	0.0223 (4)	
O13	0.39387 (13)	0.34235 (15)	0.54308 (11)	0.0277 (4)	
O14	0.32385 (14)	0.46326 (12)	0.47845 (11)	0.0246 (4)	
O15	0.23109 (12)	0.33656 (12)	0.51719 (9)	0.0197 (3)	
O16	0.06318 (13)	0.33595 (15)	0.52098 (11)	0.0278 (5)	
O17	0.13927 (14)	0.45859 (13)	0.46625 (11)	0.0251 (4)	
O18	0.06704 (13)	0.36491 (12)	0.37905 (10)	0.0213 (4)	
O19	0.16800 (13)	0.28094 (12)	0.28601 (10)	0.0227 (4)	
O20	0.24914 (12)	0.16416 (11)	0.35550 (9)	0.0191 (3)	
O21	0.33322 (14)	0.28445 (13)	0.29412 (11)	0.0253 (4)	
O22	0.24226 (12)	0.36427 (11)	0.39099 (9)	0.0171 (3)	
O23	0.14447 (11)	0.25197 (11)	0.41666 (9)	0.0164 (3)	
O24	0.33781 (11)	0.25392 (11)	0.42940 (9)	0.0172 (3)	
Mo1	0.042356 (14)	0.264056 (14)	0.348758 (11)	0.01907 (5)	
Mo2	0.142420 (14)	0.128403 (13)	0.420960 (11)	0.01805 (4)	
Mo3	0.342279 (14)	0.130828 (14)	0.434127 (12)	0.01888 (4)	
Mo4	0.454713 (15)	0.269540 (15)	0.383726 (13)	0.02328 (5)	
Mo5	0.333156 (14)	0.365314 (13)	0.475739 (11)	0.01745 (4)	
Mo6	0.134696 (14)	0.360541 (13)	0.463691 (11)	0.01669 (4)	
Mo7	0.248954 (14)	0.271359 (12)	0.343147 (10)	0.01554 (4)	
C1	0.2405 (3)	0.0776 (2)	0.1309 (2)	0.0436 (9)	
H1	0.235	0.0891	0.0844	0.052*	
C2	0.2374 (3)	0.1281 (2)	0.18283 (19)	0.0366 (8)	
H2	0.2294	0.1814	0.1794	0.044*	
C3	0.25571 (18)	0.01393 (18)	0.22512 (16)	0.0243 (5)	
C4	0.2682 (3)	−0.0505 (2)	0.2726 (2)	0.0466 (10)	
H4A	0.2705	−0.031	0.3188	0.07*	
H4B	0.3192	−0.0763	0.2623	0.07*	
H4C	0.2233	−0.0865	0.2681	0.07*	
N1	0.25288 (17)	0.00771 (16)	0.15858 (13)	0.0265 (5)	
N2	0.24786 (16)	0.08720 (15)	0.24065 (13)	0.0236 (5)	
H1N	0.255 (2)	−0.0354 (12)	0.1362 (13)	0.028*	
H2N	0.248 (2)	0.1084 (15)	0.2801 (10)	0.028*	
C5	0.4466 (3)	0.0710 (3)	0.1457 (2)	0.0472 (10)	
H5	0.4409	0.0769	0.0984	0.057*	
C6	0.4432 (3)	0.1273 (3)	0.1927 (2)	0.0429 (9)	
H6	0.4352	0.1801	0.1847	0.051*	
C7	0.46291 (19)	0.0184 (2)	0.2452 (2)	0.0338 (7)	
C8	0.4797 (3)	−0.0370 (3)	0.3006 (2)	0.0458 (10)	
H8A	0.5322	−0.0253	0.3215	0.069*	
H8B	0.4807	−0.0887	0.2822	0.069*	
H8C	0.4372	−0.0333	0.3344	0.069*	
N3	0.45995 (18)	0.00367 (19)	0.18011 (18)	0.0356 (7)	
N4	0.45352 (17)	0.09260 (18)	0.25376 (18)	0.0343 (6)	
H3N	0.457 (3)	−0.0409 (12)	0.1597 (14)	0.041*	
H4N	0.450 (3)	0.1166 (16)	0.2926 (10)	0.041*	
C9	0.4628 (3)	0.5730 (2)	0.3839 (2)	0.0417 (9)	
H9	0.4661	0.5816	0.431	0.05*	
C10	0.4672 (2)	0.6261 (2)	0.3346 (2)	0.0389 (8)	
H10	0.4739	0.6794	0.3407	0.047*	
C11	0.45236 (19)	0.5147 (2)	0.28609 (19)	0.0308 (7)	
C12	0.4469 (3)	0.4532 (3)	0.2355 (2)	0.0536 (12)	
H12A	0.4124	0.4697	0.1979	0.08*	
H12B	0.4235	0.4077	0.2561	0.08*	
H12C	0.5011	0.4413	0.219	0.08*	
N5	0.45244 (17)	0.50405 (18)	0.35196 (16)	0.0316 (6)	
N6	0.46028 (17)	0.58857 (17)	0.27414 (16)	0.0316 (6)	
H5N	0.446 (3)	0.4604 (12)	0.3735 (13)	0.038*	
H6N	0.456 (3)	0.6126 (15)	0.2346 (10)	0.038*	
C13	0.4092 (3)	0.3119 (4)	0.0588 (3)	0.0606 (14)	
H13	0.4644	0.327	0.0599	0.073*	
C14	0.3540 (3)	0.3234 (3)	0.1070 (3)	0.0549 (12)	
H14	0.363	0.3476	0.1492	0.066*	
C15	0.2921 (3)	0.2642 (2)	0.02298 (19)	0.0364 (8)	
C16	0.2296 (4)	0.2267 (3)	−0.0190 (3)	0.0564 (12)	
H16A	0.2271	0.1726	−0.0075	0.085*	
H16B	0.2433	0.2323	−0.0666	0.085*	
H16C	0.177	0.2503	−0.0108	0.085*	
N7	0.3698 (2)	0.2740 (2)	0.00784 (19)	0.0475 (9)	
N8	0.2818 (2)	0.2935 (2)	0.08359 (17)	0.0398 (7)	
H7N	0.3920 (18)	0.263 (3)	−0.0313 (13)	0.048*	
H8N	0.2341 (13)	0.301 (3)	0.1029 (17)	0.048*	
C17	0.3403 (2)	0.3084 (2)	0.75070 (18)	0.0319 (7)	
H17	0.3787	0.2916	0.7834	0.038*	
C18	0.34305 (19)	0.2954 (2)	0.68388 (18)	0.0300 (6)	
H18	0.3836	0.2681	0.6606	0.036*	
C19	0.23323 (18)	0.36274 (19)	0.70469 (15)	0.0251 (5)	
C20	0.1568 (2)	0.4053 (3)	0.6948 (2)	0.0404 (9)	
H20A	0.119	0.392	0.7305	0.061*	
H20B	0.1328	0.392	0.651	0.061*	
H20C	0.1679	0.46	0.6962	0.061*	
N9	0.27156 (17)	0.35055 (18)	0.76280 (14)	0.0292 (5)	
N10	0.27544 (16)	0.32947 (17)	0.65606 (13)	0.0260 (5)	
H9N	0.2597 (19)	0.369 (2)	0.8029 (10)	0.031*	
H10N	0.2612 (19)	0.328 (2)	0.6133 (9)	0.031*	
C21	0.1295 (2)	0.2036 (2)	0.62572 (16)	0.0294 (6)	
H21	0.0909	0.2298	0.5985	0.035*	
C22	0.12795 (19)	0.1954 (2)	0.69351 (15)	0.0289 (6)	
H22	0.0878	0.2144	0.7228	0.035*	
C23	0.23697 (19)	0.13713 (18)	0.65689 (14)	0.0241 (5)	
C24	0.3139 (2)	0.0943 (2)	0.6550 (2)	0.0356 (7)	
H24A	0.3575	0.1257	0.6739	0.053*	
H24B	0.3264	0.0813	0.6083	0.053*	
H24C	0.3088	0.0476	0.6815	0.053*	
N11	0.19766 (16)	0.16674 (17)	0.60401 (12)	0.0262 (5)	
N12	0.19571 (16)	0.15416 (17)	0.71190 (12)	0.0257 (5)	
H11N	0.2134 (19)	0.164 (2)	0.5621 (9)	0.031*	
H12N	0.2122 (19)	0.145 (2)	0.7531 (9)	0.031*	
C25	0.3331 (3)	−0.0694 (3)	0.5204 (2)	0.0543 (12)	
H25	0.3753	−0.0638	0.4888	0.065*	
C26	0.3380 (3)	−0.1023 (3)	0.5821 (2)	0.0498 (11)	
H26	0.3853	−0.1248	0.6009	0.06*	
C27	0.2140 (3)	−0.0632 (2)	0.5699 (2)	0.0439 (10)	
C28	0.1286 (4)	−0.0448 (3)	0.5815 (3)	0.0625 (14)	
H28A	0.0994	−0.0912	0.5938	0.094*	
H28B	0.1046	−0.0234	0.5404	0.094*	
H28C	0.1248	−0.0077	0.618	0.094*	
N13	0.2543 (3)	−0.0461 (2)	0.51329 (18)	0.0523 (10)	
N14	0.2641 (2)	−0.0981 (2)	0.61320 (16)	0.0421 (8)	
H13N	0.2347 (18)	−0.022 (3)	0.4779 (15)	0.05*	
C29	0.1459 (2)	0.5736 (2)	0.59154 (18)	0.0331 (7)	
H29	0.0966	0.5697	0.5669	0.04*	
C30	0.1591 (2)	0.6127 (2)	0.64964 (18)	0.0325 (7)	
H30	0.1195	0.6411	0.673	0.039*	
C31	0.2732 (2)	0.5611 (2)	0.62385 (16)	0.0289 (6)	
C32	0.3600 (2)	0.5372 (3)	0.6246 (2)	0.0472 (11)	
H32A	0.3916	0.5723	0.6526	0.071*	
H32B	0.3807	0.5378	0.5786	0.071*	
H32C	0.3645	0.4856	0.643	0.071*	
N15	0.21866 (17)	0.54082 (17)	0.57597 (14)	0.0287 (5)	
N16	0.23894 (17)	0.60467 (18)	0.66955 (14)	0.0302 (6)	
H15N	0.2266 (18)	0.511 (2)	0.5419 (15)	0.036*	
O25	0.14267 (16)	0.30433 (19)	0.14871 (12)	0.0369 (6)	
H25V	0.134 (3)	0.300 (3)	0.1909 (10)	0.055*	
H25W	0.0957 (17)	0.302 (3)	0.130 (2)	0.055*	
O26	0.4963 (2)	0.2100 (2)	0.58818 (17)	0.0519 (8)	
H26V	0.482 (4)	0.248 (2)	0.566 (3)	0.078*	
H26W	0.482 (4)	0.172 (2)	0.566 (3)	0.078*	

### Atomic displacement parameters (Å^2^)

**Table:**

	*U*^11^	*U*^22^	*U*^33^	*U*^12^	*U*^13^	*U*^23^
O1	0.0214 (9)	0.0333 (12)	0.0294 (11)	−0.0021 (8)	0.0030 (8)	0.0072 (9)
O2	0.0330 (11)	0.0339 (12)	0.0182 (9)	−0.0065 (9)	−0.0104 (8)	0.0047 (8)
O3	0.0288 (10)	0.0217 (9)	0.0183 (9)	−0.0051 (8)	−0.0051 (7)	0.0004 (7)
O4	0.0258 (10)	0.0345 (12)	0.0206 (9)	−0.0030 (9)	0.0017 (8)	0.0070 (8)
O5	0.0382 (12)	0.0218 (10)	0.0285 (11)	−0.0060 (9)	−0.0035 (9)	−0.0020 (8)
O6	0.0218 (8)	0.0208 (9)	0.0129 (7)	−0.0003 (7)	0.0016 (6)	0.0024 (6)
O7	0.0255 (10)	0.0328 (12)	0.0268 (11)	0.0018 (9)	−0.0034 (8)	0.0067 (9)
O8	0.0375 (12)	0.0221 (10)	0.0247 (10)	0.0050 (9)	0.0080 (9)	0.0008 (8)
O9	0.0295 (10)	0.0258 (10)	0.0253 (10)	0.0044 (8)	0.0119 (8)	0.0025 (8)
O10	0.0240 (11)	0.0398 (14)	0.0452 (15)	0.0009 (10)	−0.0018 (10)	0.0059 (12)
O11	0.0371 (13)	0.0433 (15)	0.0379 (14)	0.0011 (11)	0.0227 (11)	0.0077 (11)
O12	0.0239 (9)	0.0206 (9)	0.0223 (9)	−0.0029 (7)	0.0041 (7)	0.0025 (7)
O13	0.0233 (10)	0.0389 (13)	0.0206 (9)	−0.0016 (9)	−0.0060 (8)	0.0031 (9)
O14	0.0312 (10)	0.0204 (9)	0.0222 (9)	−0.0040 (8)	−0.0020 (8)	−0.0034 (7)
O15	0.0219 (9)	0.0257 (9)	0.0116 (7)	−0.0001 (7)	−0.0011 (6)	0.0004 (7)
O16	0.0256 (10)	0.0402 (13)	0.0177 (9)	0.0037 (9)	0.0059 (8)	0.0036 (8)
O17	0.0310 (11)	0.0228 (10)	0.0215 (9)	0.0033 (8)	−0.0038 (8)	−0.0032 (7)
O18	0.0242 (9)	0.0206 (9)	0.0189 (9)	0.0013 (7)	−0.0031 (7)	0.0008 (7)
O19	0.0306 (10)	0.0237 (9)	0.0137 (8)	−0.0029 (8)	−0.0038 (7)	0.0018 (7)
O20	0.0283 (9)	0.0160 (8)	0.0129 (7)	−0.0013 (7)	0.0032 (7)	−0.0007 (6)
O21	0.0321 (11)	0.0255 (10)	0.0186 (9)	0.0004 (8)	0.0110 (8)	0.0020 (7)
O22	0.0229 (8)	0.0167 (8)	0.0117 (7)	−0.0007 (6)	0.0003 (6)	−0.0007 (6)
O23	0.0187 (8)	0.0189 (8)	0.0117 (7)	−0.0014 (6)	0.0007 (6)	0.0008 (6)
O24	0.0198 (8)	0.0175 (8)	0.0143 (7)	0.0004 (6)	0.0029 (6)	0.0014 (6)
Mo1	0.01912 (9)	0.02215 (10)	0.01584 (9)	−0.00268 (8)	−0.00351 (7)	0.00174 (7)
Mo2	0.02159 (10)	0.01762 (9)	0.01494 (9)	−0.00348 (7)	−0.00019 (7)	0.00209 (7)
Mo3	0.02075 (10)	0.01832 (10)	0.01763 (9)	0.00146 (8)	0.00352 (7)	0.00293 (7)
Mo4	0.01970 (10)	0.02472 (11)	0.02561 (11)	0.00006 (8)	0.00868 (8)	0.00360 (9)
Mo5	0.01950 (9)	0.01916 (10)	0.01367 (9)	−0.00115 (7)	−0.00133 (7)	−0.00076 (7)
Mo6	0.01876 (9)	0.01945 (10)	0.01188 (8)	0.00120 (7)	0.00054 (7)	−0.00103 (7)
Mo7	0.02116 (9)	0.01513 (9)	0.01035 (8)	−0.00110 (7)	0.00168 (7)	−0.00035 (6)
C1	0.069 (3)	0.0360 (19)	0.0254 (16)	0.0063 (19)	−0.0037 (17)	−0.0048 (14)
C2	0.054 (2)	0.0268 (15)	0.0291 (16)	0.0069 (15)	−0.0016 (15)	−0.0060 (12)
C3	0.0225 (12)	0.0242 (13)	0.0263 (13)	0.0004 (10)	0.0014 (10)	−0.0075 (10)
C4	0.076 (3)	0.0280 (17)	0.036 (2)	−0.0038 (18)	0.002 (2)	−0.0050 (15)
N1	0.0293 (12)	0.0281 (12)	0.0222 (11)	0.0017 (10)	0.0000 (9)	−0.0128 (9)
N2	0.0248 (11)	0.0248 (11)	0.0211 (11)	0.0002 (9)	0.0014 (9)	−0.0092 (9)
C5	0.052 (2)	0.051 (3)	0.038 (2)	0.000 (2)	−0.0123 (18)	−0.0113 (18)
C6	0.047 (2)	0.041 (2)	0.041 (2)	0.0030 (17)	−0.0089 (17)	−0.0061 (17)
C7	0.0215 (13)	0.0335 (17)	0.047 (2)	−0.0041 (12)	0.0096 (13)	−0.0113 (14)
C8	0.045 (2)	0.046 (2)	0.046 (2)	0.0079 (18)	0.0100 (18)	−0.0030 (18)
N3	0.0256 (13)	0.0373 (16)	0.0439 (17)	−0.0015 (11)	0.0025 (12)	−0.0179 (13)
N4	0.0231 (12)	0.0330 (15)	0.0469 (18)	−0.0027 (11)	0.0027 (11)	−0.0127 (13)
C9	0.050 (2)	0.037 (2)	0.038 (2)	−0.0067 (17)	0.0036 (17)	0.0054 (15)
C10	0.043 (2)	0.0313 (17)	0.043 (2)	−0.0031 (15)	0.0023 (16)	0.0063 (15)
C11	0.0230 (13)	0.0311 (15)	0.0380 (17)	−0.0034 (11)	−0.0103 (12)	0.0118 (13)
C12	0.073 (3)	0.040 (2)	0.047 (2)	−0.004 (2)	−0.020 (2)	0.0017 (19)
N5	0.0250 (12)	0.0308 (14)	0.0389 (16)	−0.0056 (10)	−0.0032 (11)	0.0133 (12)
N6	0.0261 (12)	0.0306 (14)	0.0380 (15)	−0.0036 (10)	−0.0054 (11)	0.0120 (12)
C13	0.037 (2)	0.077 (4)	0.068 (3)	0.002 (2)	0.012 (2)	−0.008 (3)
C14	0.045 (2)	0.070 (3)	0.050 (3)	−0.003 (2)	0.005 (2)	−0.020 (2)
C15	0.047 (2)	0.0326 (17)	0.0296 (16)	0.0093 (15)	0.0081 (14)	0.0016 (13)
C16	0.077 (4)	0.044 (3)	0.047 (3)	0.002 (2)	−0.011 (2)	−0.005 (2)
N7	0.053 (2)	0.052 (2)	0.0381 (18)	0.0154 (17)	0.0202 (16)	0.0027 (16)
N8	0.0393 (16)	0.0454 (19)	0.0350 (16)	0.0044 (14)	0.0116 (13)	−0.0041 (14)
C17	0.0242 (13)	0.0393 (18)	0.0321 (16)	0.0027 (12)	−0.0053 (12)	0.0007 (13)
C18	0.0220 (13)	0.0359 (17)	0.0322 (16)	0.0035 (12)	0.0030 (11)	−0.0006 (13)
C19	0.0228 (12)	0.0309 (15)	0.0215 (12)	0.0022 (11)	0.0018 (10)	−0.0001 (10)
C20	0.0333 (17)	0.048 (2)	0.040 (2)	0.0146 (16)	−0.0022 (14)	0.0057 (17)
N9	0.0293 (13)	0.0380 (15)	0.0203 (11)	0.0040 (11)	−0.0012 (9)	−0.0007 (10)
N10	0.0266 (12)	0.0338 (13)	0.0177 (10)	−0.0006 (10)	0.0024 (9)	−0.0001 (9)
C21	0.0277 (14)	0.0417 (18)	0.0190 (12)	0.0009 (13)	0.0000 (10)	0.0026 (12)
C22	0.0268 (13)	0.0415 (18)	0.0187 (12)	−0.0004 (12)	0.0069 (10)	0.0002 (11)
C23	0.0288 (13)	0.0282 (14)	0.0155 (11)	−0.0015 (11)	0.0017 (10)	−0.0007 (9)
C24	0.0362 (17)	0.0365 (18)	0.0343 (17)	0.0089 (14)	0.0056 (14)	0.0012 (14)
N11	0.0289 (12)	0.0374 (14)	0.0124 (9)	−0.0002 (10)	0.0027 (8)	0.0008 (9)
N12	0.0293 (12)	0.0347 (14)	0.0132 (9)	−0.0017 (10)	0.0013 (8)	0.0037 (9)
C25	0.081 (3)	0.039 (2)	0.043 (2)	0.008 (2)	0.026 (2)	0.0121 (18)
C26	0.066 (3)	0.042 (2)	0.042 (2)	0.003 (2)	0.013 (2)	0.0089 (18)
C27	0.074 (3)	0.0266 (16)	0.0306 (17)	−0.0031 (18)	0.0014 (18)	0.0015 (13)
C28	0.075 (4)	0.054 (3)	0.058 (3)	−0.001 (3)	−0.006 (3)	0.005 (2)
N13	0.095 (3)	0.0330 (17)	0.0288 (16)	0.0064 (19)	0.0075 (18)	0.0112 (13)
N14	0.065 (2)	0.0348 (16)	0.0260 (14)	−0.0038 (15)	0.0053 (14)	0.0052 (12)
C29	0.0285 (15)	0.0419 (19)	0.0286 (15)	0.0052 (13)	−0.0094 (12)	−0.0013 (13)
C30	0.0307 (15)	0.0369 (17)	0.0298 (15)	0.0115 (13)	−0.0028 (12)	−0.0057 (13)
C31	0.0290 (14)	0.0355 (16)	0.0222 (13)	0.0043 (12)	−0.0059 (11)	−0.0094 (12)
C32	0.0322 (18)	0.068 (3)	0.041 (2)	0.0155 (19)	−0.0086 (15)	−0.019 (2)
N15	0.0315 (13)	0.0327 (14)	0.0217 (11)	0.0048 (11)	−0.0046 (10)	−0.0109 (10)
N16	0.0302 (13)	0.0364 (15)	0.0240 (12)	0.0088 (11)	−0.0057 (10)	−0.0092 (11)
O25	0.0344 (12)	0.0563 (17)	0.0199 (10)	0.0001 (12)	0.0025 (9)	0.0003 (11)
O26	0.066 (2)	0.0466 (18)	0.0422 (17)	−0.0038 (16)	−0.0272 (15)	0.0040 (14)

### Geometric parameters (Å, º)

**Table:**

O1—Mo1	1.724 (2)	C11—N6	1.328 (4)
O2—Mo1	1.725 (2)	C11—C12	1.479 (6)
O3—Mo1	1.945 (2)	C12—H12A	0.98
O3—Mo2	1.971 (2)	C12—H12B	0.98
O4—Mo2	1.707 (2)	C12—H12C	0.98
O5—Mo2	1.725 (2)	N5—H5N	0.886 (17)
O6—Mo3	1.9528 (19)	N6—H6N	0.895 (17)
O6—Mo2	1.953 (2)	C13—C14	1.347 (7)
O7—Mo3	1.715 (2)	C13—N7	1.372 (7)
O8—Mo3	1.716 (2)	C13—H13	0.95
O9—Mo4	1.939 (2)	C14—N8	1.379 (6)
O9—Mo3	1.987 (2)	C14—H14	0.95
O10—Mo4	1.709 (3)	C15—N8	1.324 (5)
O11—Mo4	1.715 (2)	C15—N7	1.333 (5)
O12—Mo4	1.932 (2)	C15—C16	1.476 (6)
O12—Mo5	1.983 (2)	C16—H16A	0.98
O13—Mo5	1.713 (2)	C16—H16B	0.98
O14—Mo5	1.730 (2)	C16—H16C	0.98
O15—Mo6	1.9521 (19)	N7—H7N	0.887 (17)
O15—Mo5	1.953 (2)	N8—H8N	0.893 (17)
O16—Mo6	1.707 (2)	C17—C18	1.350 (5)
O17—Mo6	1.726 (2)	C17—N9	1.380 (4)
O18—Mo1	1.916 (2)	C17—H17	0.95
O18—Mo6	2.012 (2)	C18—N10	1.379 (4)
O19—Mo7	1.754 (2)	C18—H18	0.95
O19—Mo1	2.453 (2)	C19—N9	1.329 (4)
O20—Mo7	1.901 (2)	C19—N10	1.334 (4)
O20—Mo3	2.259 (2)	C19—C20	1.480 (5)
O20—Mo2	2.292 (2)	C20—H20A	0.98
O21—Mo7	1.725 (2)	C20—H20B	0.98
O22—Mo7	1.8945 (19)	C20—H20C	0.98
O22—Mo5	2.2453 (19)	N9—H9N	0.886 (17)
O22—Mo6	2.3057 (19)	N10—H10N	0.880 (17)
O23—Mo6	2.1329 (19)	C21—C22	1.356 (4)
O23—Mo1	2.1604 (19)	C21—N11	1.374 (4)
O23—Mo2	2.1748 (19)	C21—H21	0.95
O23—Mo7	2.3011 (18)	C22—N12	1.380 (4)
O24—Mo4	2.1624 (19)	C22—H22	0.95
O24—Mo5	2.1665 (19)	C23—N12	1.330 (4)
O24—Mo3	2.1677 (19)	C23—N11	1.336 (4)
O24—Mo7	2.2665 (19)	C23—C24	1.479 (5)
Mo1—Mo6	3.2178 (3)	C24—H24A	0.98
Mo4—Mo5	3.2105 (3)	C24—H24B	0.98
C1—C2	1.362 (5)	C24—H24C	0.98
C1—N1	1.362 (5)	N11—H11N	0.877 (17)
C1—H1	0.95	N12—H12N	0.876 (17)
C2—N2	1.365 (5)	C25—C26	1.357 (6)
C2—H2	0.95	C25—N13	1.371 (7)
C3—N1	1.328 (4)	C25—H25	0.95
C3—N2	1.332 (4)	C26—N14	1.377 (6)
C3—C4	1.488 (5)	C26—H26	0.95
C4—H4A	0.98	C27—N14	1.338 (6)
C4—H4B	0.98	C27—N13	1.348 (6)
C4—H4C	0.98	C27—C28	1.470 (8)
N1—H1N	0.881 (17)	C28—H28A	0.98
N2—H2N	0.868 (17)	C28—H28B	0.98
C5—C6	1.363 (6)	C28—H28C	0.98
C5—N3	1.384 (6)	N13—H13N	0.880 (19)
C5—H5	0.95	C29—C30	1.360 (5)
C6—N4	1.367 (6)	C29—N15	1.372 (4)
C6—H6	0.95	C29—H29	0.95
C7—N3	1.321 (5)	C30—N16	1.380 (4)
C7—N4	1.325 (5)	C30—H30	0.95
C7—C8	1.493 (6)	C31—N16	1.321 (4)
C8—H8A	0.98	C31—N15	1.352 (4)
C8—H8B	0.98	C31—C32	1.495 (5)
C8—H8C	0.98	C32—H32A	0.98
N3—H3N	0.884 (17)	C32—H32B	0.98
N4—H4N	0.884 (17)	C32—H32C	0.98
C9—C10	1.357 (6)	N15—H15N	0.867 (19)
C9—N5	1.378 (5)	O25—H25V	0.857 (19)
C9—H9	0.95	O25—H25W	0.856 (18)
C10—N6	1.375 (5)	O26—H26V	0.842 (19)
C10—H10	0.95	O26—H26W	0.843 (19)
C11—N5	1.323 (5)		
			
Mo1—O3—Mo2	111.13 (10)	C3—C4—H4A	109.5
Mo3—O6—Mo2	115.96 (9)	C3—C4—H4B	109.5
Mo4—O9—Mo3	110.89 (11)	H4A—C4—H4B	109.5
Mo4—O12—Mo5	110.14 (10)	C3—C4—H4C	109.5
Mo6—O15—Mo5	114.70 (9)	H4A—C4—H4C	109.5
Mo1—O18—Mo6	110.01 (10)	H4B—C4—H4C	109.5
Mo7—O19—Mo1	107.64 (9)	C3—N1—C1	109.4 (3)
Mo7—O20—Mo3	110.29 (9)	C3—N1—H1N	125.1 (19)
Mo7—O20—Mo2	110.20 (9)	C1—N1—H1N	125.4 (19)
Mo3—O20—Mo2	93.36 (7)	C3—N2—C2	109.0 (3)
Mo7—O22—Mo5	109.98 (9)	C3—N2—H2N	128.7 (19)
Mo7—O22—Mo6	109.89 (9)	C2—N2—H2N	122.3 (19)
Mo5—O22—Mo6	92.51 (7)	C6—C5—N3	106.9 (4)
Mo6—O23—Mo1	97.09 (8)	C6—C5—H5	126.6
Mo6—O23—Mo2	151.14 (9)	N3—C5—H5	126.6
Mo1—O23—Mo2	96.34 (7)	C5—C6—N4	106.2 (4)
Mo6—O23—Mo7	101.96 (8)	C5—C6—H6	126.9
Mo1—O23—Mo7	100.09 (7)	N4—C6—H6	126.9
Mo2—O23—Mo7	100.67 (7)	N3—C7—N4	108.4 (4)
Mo4—O24—Mo5	95.74 (8)	N3—C7—C8	126.8 (4)
Mo4—O24—Mo3	96.60 (8)	N4—C7—C8	124.7 (4)
Mo5—O24—Mo3	152.34 (9)	C7—C8—H8A	109.5
Mo4—O24—Mo7	103.86 (7)	C7—C8—H8B	109.5
Mo5—O24—Mo7	100.00 (8)	H8A—C8—H8B	109.5
Mo3—O24—Mo7	100.92 (8)	C7—C8—H8C	109.5
O1—Mo1—O2	104.12 (11)	H8A—C8—H8C	109.5
O1—Mo1—O18	98.92 (11)	H8B—C8—H8C	109.5
O2—Mo1—O18	102.57 (10)	C7—N3—C5	108.7 (3)
O1—Mo1—O3	96.55 (11)	C7—N3—H3N	129 (2)
O2—Mo1—O3	100.84 (10)	C5—N3—H3N	122 (2)
O18—Mo1—O3	147.73 (9)	C7—N4—C6	109.9 (3)
O1—Mo1—O23	106.54 (9)	C7—N4—H4N	126 (2)
O2—Mo1—O23	149.29 (10)	C6—N4—H4N	124 (2)
O18—Mo1—O23	74.47 (8)	C10—C9—N5	106.3 (4)
O3—Mo1—O23	74.03 (8)	C10—C9—H9	126.9
O1—Mo1—O19	176.53 (9)	N5—C9—H9	126.9
O2—Mo1—O19	78.58 (10)	C9—C10—N6	107.2 (4)
O18—Mo1—O19	82.49 (8)	C9—C10—H10	126.4
O3—Mo1—O19	80.71 (8)	N6—C10—H10	126.4
O23—Mo1—O19	70.72 (7)	N5—C11—N6	108.4 (3)
O1—Mo1—Mo6	95.01 (8)	N5—C11—C12	124.7 (3)
O2—Mo1—Mo6	137.30 (8)	N6—C11—C12	126.8 (4)
O18—Mo1—Mo6	35.98 (6)	C11—C12—H12A	109.5
O3—Mo1—Mo6	114.62 (6)	C11—C12—H12B	109.5
O23—Mo1—Mo6	41.13 (5)	H12A—C12—H12B	109.5
O19—Mo1—Mo6	84.26 (5)	C11—C12—H12C	109.5
O4—Mo2—O5	105.64 (12)	H12A—C12—H12C	109.5
O4—Mo2—O6	96.30 (9)	H12B—C12—H12C	109.5
O5—Mo2—O6	97.94 (10)	C11—N5—C9	109.3 (3)
O4—Mo2—O3	102.62 (10)	C11—N5—H5N	127.0 (19)
O5—Mo2—O3	95.05 (10)	C9—N5—H5N	123.7 (19)
O6—Mo2—O3	153.21 (8)	C11—N6—C10	108.7 (3)
O4—Mo2—O23	93.82 (10)	C11—N6—H6N	127.8 (19)
O5—Mo2—O23	159.22 (10)	C10—N6—H6N	123.1 (19)
O6—Mo2—O23	86.87 (8)	C14—C13—N7	106.2 (4)
O3—Mo2—O23	73.22 (8)	C14—C13—H13	126.9
O4—Mo2—O20	161.90 (10)	N7—C13—H13	126.9
O5—Mo2—O20	90.12 (10)	C13—C14—N8	107.0 (4)
O6—Mo2—O20	72.28 (7)	C13—C14—H14	126.5
O3—Mo2—O20	84.40 (8)	N8—C14—H14	126.5
O23—Mo2—O20	72.01 (7)	N8—C15—N7	106.8 (4)
O8—Mo3—O7	104.86 (12)	N8—C15—C16	126.4 (4)
O8—Mo3—O6	100.55 (10)	N7—C15—C16	126.8 (4)
O7—Mo3—O6	96.34 (10)	C15—C16—H16A	109.5
O8—Mo3—O9	92.85 (10)	C15—C16—H16B	109.5
O7—Mo3—O9	100.23 (11)	H16A—C16—H16B	109.5
O6—Mo3—O9	155.25 (9)	C15—C16—H16C	109.5
O8—Mo3—O24	155.03 (9)	H16A—C16—H16C	109.5
O7—Mo3—O24	97.84 (10)	H16B—C16—H16C	109.5
O6—Mo3—O24	86.96 (8)	C15—N7—C13	110.3 (4)
O9—Mo3—O24	72.72 (8)	C15—N7—H7N	125 (2)
O8—Mo3—O20	87.48 (10)	C13—N7—H7N	124 (2)
O7—Mo3—O20	165.23 (10)	C15—N8—C14	109.7 (4)
O6—Mo3—O20	73.05 (7)	C15—N8—H8N	125 (2)
O9—Mo3—O20	86.99 (9)	C14—N8—H8N	124 (2)
O24—Mo3—O20	71.84 (7)	C18—C17—N9	107.4 (3)
O10—Mo4—O11	106.30 (14)	C18—C17—H17	126.3
O10—Mo4—O12	99.66 (12)	N9—C17—H17	126.3
O11—Mo4—O12	101.36 (12)	C17—C18—N10	106.6 (3)
O10—Mo4—O9	100.30 (12)	C17—C18—H18	126.7
O11—Mo4—O9	99.31 (12)	N10—C18—H18	126.7
O12—Mo4—O9	145.80 (9)	N9—C19—N10	108.1 (3)
O10—Mo4—O24	106.87 (10)	N9—C19—C20	126.5 (3)
O11—Mo4—O24	146.81 (11)	N10—C19—C20	125.3 (3)
O12—Mo4—O24	74.02 (8)	C19—C20—H20A	109.5
O9—Mo4—O24	73.74 (8)	C19—C20—H20B	109.5
O10—Mo4—Mo5	93.77 (10)	H20A—C20—H20B	109.5
O11—Mo4—Mo5	135.81 (10)	C19—C20—H20C	109.5
O12—Mo4—Mo5	35.45 (6)	H20A—C20—H20C	109.5
O9—Mo4—Mo5	115.58 (6)	H20B—C20—H20C	109.5
O24—Mo4—Mo5	42.18 (5)	C19—N9—C17	108.7 (3)
O13—Mo5—O14	105.18 (11)	C19—N9—H9N	128.0 (19)
O13—Mo5—O15	96.34 (10)	C17—N9—H9N	123.0 (19)
O14—Mo5—O15	99.61 (10)	C19—N10—C18	109.2 (3)
O13—Mo5—O12	99.85 (10)	C19—N10—H10N	125.4 (19)
O14—Mo5—O12	92.28 (10)	C18—N10—H10N	125.4 (19)
O15—Mo5—O12	156.65 (9)	C22—C21—N11	106.6 (3)
O13—Mo5—O24	95.51 (10)	C22—C21—H21	126.7
O14—Mo5—O24	156.48 (9)	N11—C21—H21	126.7
O15—Mo5—O24	88.88 (8)	C21—C22—N12	107.2 (3)
O12—Mo5—O24	72.96 (8)	C21—C22—H22	126.4
O13—Mo5—O22	165.08 (10)	N12—C22—H22	126.4
O14—Mo5—O22	88.42 (9)	N12—C23—N11	108.1 (3)
O15—Mo5—O22	74.97 (7)	N12—C23—C24	125.8 (3)
O12—Mo5—O22	85.42 (8)	N11—C23—C24	126.1 (3)
O24—Mo5—O22	72.54 (7)	C23—C24—H24A	109.5
O13—Mo5—Mo4	87.53 (8)	C23—C24—H24B	109.5
O14—Mo5—Mo4	126.61 (8)	H24A—C24—H24B	109.5
O15—Mo5—Mo4	130.86 (6)	C23—C24—H24C	109.5
O12—Mo5—Mo4	34.41 (6)	H24A—C24—H24C	109.5
O24—Mo5—Mo4	42.08 (5)	H24B—C24—H24C	109.5
O22—Mo5—Mo4	89.18 (5)	C23—N11—C21	109.3 (2)
O16—Mo6—O17	105.29 (12)	C23—N11—H11N	125.3 (18)
O16—Mo6—O15	98.56 (10)	C21—N11—H11N	125.4 (18)
O17—Mo6—O15	99.46 (10)	C23—N12—C22	108.8 (2)
O16—Mo6—O18	100.69 (10)	C23—N12—H12N	124.9 (19)
O17—Mo6—O18	90.59 (9)	C22—N12—H12N	125.9 (19)
O15—Mo6—O18	155.08 (8)	C26—C25—N13	105.5 (4)
O16—Mo6—O23	97.03 (10)	C26—C25—H25	127.2
O17—Mo6—O23	154.60 (9)	N13—C25—H25	127.2
O15—Mo6—O23	88.86 (8)	C25—C26—N14	109.8 (5)
O18—Mo6—O23	73.23 (8)	C25—C26—H26	125.1
O16—Mo6—O22	166.13 (10)	N14—C26—H26	125.1
O17—Mo6—O22	87.49 (9)	N14—C27—N13	109.4 (4)
O15—Mo6—O22	73.58 (7)	N14—C27—C28	126.1 (4)
O18—Mo6—O22	84.24 (8)	N13—C27—C28	124.5 (4)
O23—Mo6—O22	71.80 (7)	C27—C28—H28A	109.5
O16—Mo6—Mo1	90.80 (8)	C27—C28—H28B	109.5
O17—Mo6—Mo1	124.59 (7)	H28A—C28—H28B	109.5
O15—Mo6—Mo1	130.62 (6)	C27—C28—H28C	109.5
O18—Mo6—Mo1	34.01 (6)	H28A—C28—H28C	109.5
O23—Mo6—Mo1	41.78 (5)	H28B—C28—H28C	109.5
O22—Mo6—Mo1	86.14 (5)	C27—N13—C25	108.9 (4)
O21—Mo7—O19	103.66 (11)	C27—N13—H13N	126 (2)
O21—Mo7—O22	102.76 (10)	C25—N13—H13N	125 (2)
O19—Mo7—O22	101.19 (9)	C27—N14—C26	106.3 (4)
O21—Mo7—O20	101.84 (10)	C30—C29—N15	105.7 (3)
O19—Mo7—O20	100.32 (9)	C30—C29—H29	127.1
O22—Mo7—O20	142.21 (8)	N15—C29—H29	127.1
O21—Mo7—O24	85.71 (9)	C29—C30—N16	109.6 (3)
O19—Mo7—O24	170.59 (8)	C29—C30—H30	125.2
O22—Mo7—O24	77.15 (8)	N16—C30—H30	125.2
O20—Mo7—O24	76.58 (8)	N16—C31—N15	110.4 (3)
O21—Mo7—O23	174.78 (9)	N16—C31—C32	125.2 (3)
O19—Mo7—O23	81.55 (8)	N15—C31—C32	124.4 (3)
O22—Mo7—O23	76.12 (8)	C31—C32—H32A	109.5
O20—Mo7—O23	76.77 (8)	C31—C32—H32B	109.5
O24—Mo7—O23	89.07 (6)	H32A—C32—H32B	109.5
C2—C1—N1	106.8 (3)	C31—C32—H32C	109.5
C2—C1—H1	126.6	H32A—C32—H32C	109.5
N1—C1—H1	126.6	H32B—C32—H32C	109.5
C1—C2—N2	106.9 (3)	C31—N15—C29	108.1 (3)
C1—C2—H2	126.6	C31—N15—H15N	127.1 (19)
N2—C2—H2	126.6	C29—N15—H15N	124.8 (19)
N1—C3—N2	108.0 (3)	C31—N16—C30	106.1 (3)
N1—C3—C4	125.0 (3)	H25V—O25—H25W	105 (3)
N2—C3—C4	127.0 (3)	H26V—O26—H26W	107 (4)

### Hydrogen-bond geometry (Å, º)

**Table:**

*D*—H···*A*	*D*—H	H···*A*	*D*···*A*	*D*—H···*A*
N1—H1*N*···O22^i^	0.88 (2)	1.85 (2)	2.710 (3)	167 (2)
N2—H2*N*···O20	0.87 (2)	1.79 (2)	2.655 (3)	172 (2)
N3—H3*N*···O18^i^	0.88 (2)	1.87 (2)	2.743 (4)	171 (5)
N4—H4*N*···O9	0.88 (2)	1.79 (2)	2.662 (4)	171 (5)
N5—H5*N*···O12	0.89 (2)	1.77 (3)	2.644 (4)	168 (4)
N6—H6*N*···O3^ii^	0.90 (2)	1.80 (3)	2.684 (4)	168 (5)
N7—H7*N*···O1^iii^	0.89 (3)	1.83 (3)	2.697 (4)	167 (3)
N8—H8*N*···O25	0.89 (3)	1.78 (3)	2.659 (4)	172 (5)
N9—H9*N*···N14^iv^	0.89 (2)	1.81 (2)	2.698 (4)	177 (3)
N10—H10*N*···O15	0.88 (2)	1.98 (2)	2.852 (3)	174 (3)
N11—H11*N*···O6	0.88 (2)	1.78 (2)	2.636 (3)	166 (3)
N12—H12*N*···N16^v^	0.88 (2)	1.87 (2)	2.725 (4)	165 (3)
N13—H13*N*···O5	0.88 (4)	2.32 (4)	3.186 (5)	167 (3)
N13—H13*N*···O8	0.88 (4)	2.55 (4)	3.043 (5)	116 (2)
N15—H15*N*···O14	0.87 (3)	2.22 (3)	2.953 (4)	142 (3)
N15—H15*N*···O17	0.87 (3)	2.27 (3)	2.917 (4)	132 (3)
O25—H25*V*···O19	0.86 (2)	2.00 (2)	2.788 (3)	154 (5)
O25—H25*W*···O26^vi^	0.86 (3)	1.85 (3)	2.702 (4)	176 (5)
O26—H26*V*···O10	0.83 (4)	2.50 (6)	2.999 (4)	119 (5)
O26—H26*V*···O13	0.83 (4)	2.25 (5)	3.009 (4)	151 (6)
O26—H26*W*···O7	0.83 (4)	1.99 (6)	2.737 (4)	149 (6)
C4—H4*A*···O8	0.98	2.35	3.228 (5)	149
C8—H8*B*···O2^i^	0.98	2.54	3.517 (6)	176
C8—H8*C*···O8	0.98	2.49	3.386 (5)	152
C10—H10···O26^vii^	0.95	2.45	3.318 (5)	151
C12—H12*A*···O5^ii^	0.98	2.44	3.407 (5)	167
C13—H13···O4^iii^	0.95	2.50	3.297 (6)	141
C17—H17···O2^viii^	0.95	2.46	3.154 (4)	130
C18—H18···O26	0.95	2.58	3.522 (5)	172
C21—H21···O16	0.95	2.46	3.302 (4)	147
C22—H22···O11^ix^	0.95	2.22	3.113 (4)	156
C24—H24*B*···O7	0.98	2.50	3.346 (5)	145
C25—H25···O8	0.95	2.59	3.055 (5)	110
C32—H32*B*···O14	0.98	2.55	3.234 (5)	126

Symmetry codes: (i) −*x*+1/2, *y*−1/2, −*z*+1/2; (ii) −*x*+1/2, *y*+1/2, −*z*+1/2; (iii) *x*+1/2, −*y*+1/2, *z*−1/2; (iv) −*x*+1/2, *y*+1/2, −*z*+3/2; (v) −*x*+1/2, *y*−1/2, −*z*+3/2; (vi) *x*−1/2, −*y*+1/2, *z*−1/2; (vii) −*x*+1, −*y*+1, −*z*+1; (viii) *x*+1/2, −*y*+1/2, *z*+1/2; (ix) *x*−1/2, −*y*+1/2, *z*+1/2.
