# Supplementary figures and images for: Synthesis and crystal structure of a new hybrid organic–inorganic material containing neutral mol­ecules, cations and hepta­molybdate anions
Source: Acta Crystallogr E Crystallogr Commun. 2019 Jun 21;75(Pt 7):1001–4. doi: 10.1107/S2056989019008454 (PMC6659346; doi:10.1107/S2056989019008454)

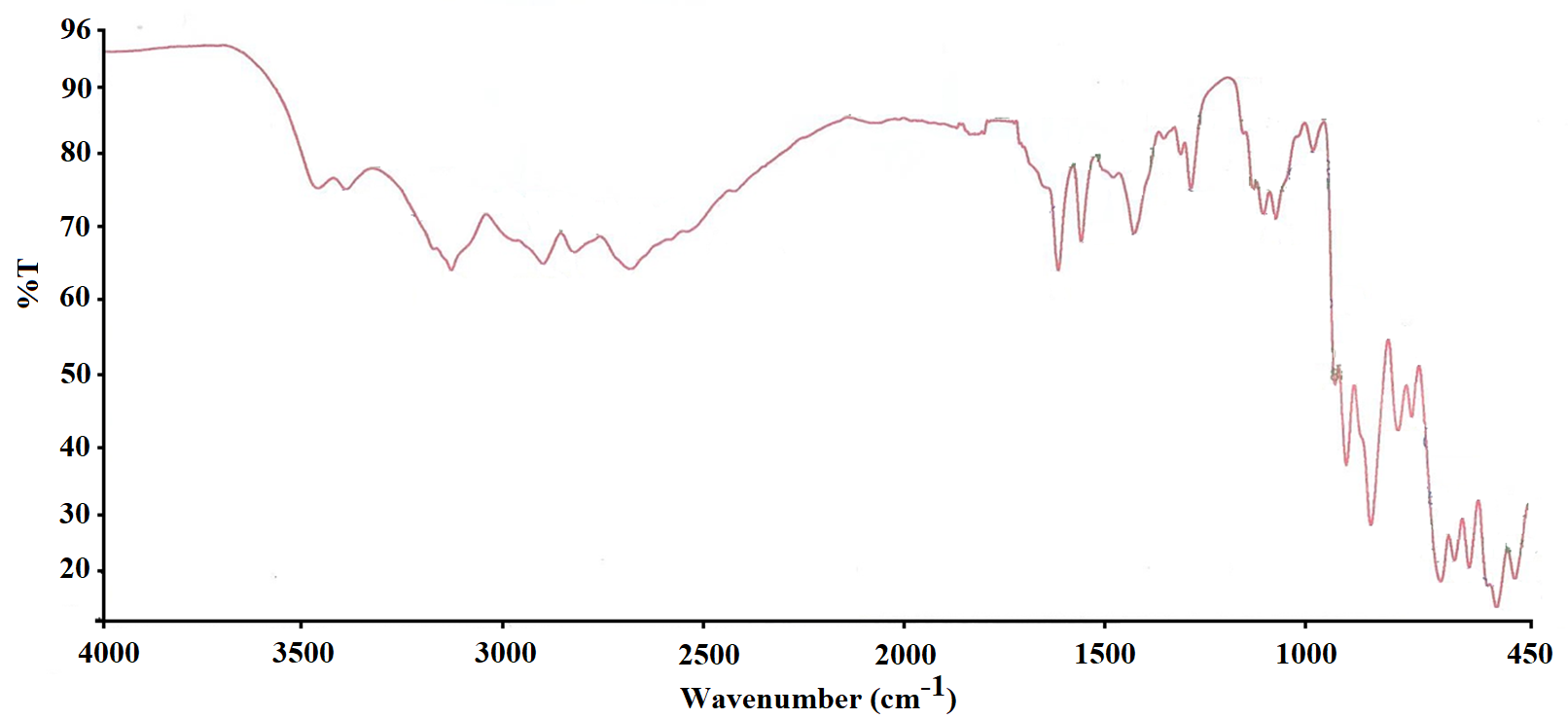

Supplement: Supplementary file 3 [file e-75-01001-sup3.tif]
